# The Predatory Myxobacterium *Citreicoccus inhibens* gen. nov. sp. nov. Showed Antifungal Activity and Bacteriolytic Property against Phytopathogens

**DOI:** 10.3390/microorganisms9102137

**Published:** 2021-10-12

**Authors:** Yang Zhou, Shuoxing Yi, Yi Zang, Qing Yao, Honghui Zhu

**Affiliations:** 1 Guangdong Provincial Key Laboratory of Microbial Culture Collection and Application, State Key Laboratory of Applied Microbiology Southern China, Guangdong Microbial Culture Collection Center (GDMCC), Institute of Microbiology, Guangdong Academy of Sciences, Guangzhou 510070, China; zhouyang@gdim.cn (Y.Z.); yisx@stu.scau.edu.cn (S.Y.); zangyi2003hunan@hotmail.com (Y.Z.); 2 College of Plant Protection, South China Agricultural University, Guangzhou 510642, China; 3 Center for Litchi, Guangdong Province Key Laboratory of Microbial Signals and Disease Control, Guangdong Engineering Research Center for Grass Science, Guangdong Engineering, College of Horticulture, South China Agricultural University, Guangzhou 510642, China

**Keywords:** *Citreicoccus inhibens* M34, predation, antifungal compounds, lytic proteins, biocontrol agent

## Abstract

The application and promotion of biological control agents are limited because of poor efficacy and unstable performance in the field. Screening microorganisms with high antagonistic activity, effective adaptability, and high field-survival should be prospected. Myxobacteria are soil predatory bacteria with wide adaptability, which are considered as good antagonists. Here, we report a myxobacterium strain M34 isolated from subtropical forest soil in South China using the *Escherichia coli* baiting method. Based on the morphological observation, physiological test, biochemical characteristics, 16S rRNA gene sequence, and genomic data, strain M34 was identified as a novel genus and novel species, representing a new clade of Myxococcaceae, for which the name *Citreicoccus inhibens* gen. nov. sp. nov. is proposed (type strain M34^T^ = GDMCC 1.2275^T^ = KCTC 82453^T^). The typical features of M34, including fruiting body formation and extracellular fibrillar interconnection, indicated by microscopic observations, contributed to cell adaption in different environments. Furthermore, the strain showed antifungal activity against phytopathogenic fungi and predatory activity to both Gram-negative and Gram-positive phytopathogenic bacteria. The bioprotective mechanisms are attributed to the presence of pyrrolnitrin and derivative with antifungal activity and the extracellular proteins with lytic activity against pathogenic bacteria. Due to its multiple beneficial traits, strain M34 has the potential to be developed into a versatile biocontrol agent for the management of both fungal and bacterial phytopathogens.

## 1. Introduction

Crop yield and quality are seriously affected by plant diseases. About 90% of the known infectious diseases of plants are caused by fungi and bacteria [1,2]. According to the data from Secretariat of the International Plant Protection Convention at the FAO, the amount of crop loss caused by plant disease was 14.1% [3]. To control plant diseases, agricultural crops are treated with chemicals in most cases. Although most of the chemical agents show high efficiency and persistence, many of them are toxic, harmful to environment, and sometimes even facing the risk of acquired resistance [4,5]. Thus, the development of efficient and eco-friendly alternatives focused on the elimination or reduction of synthetic chemicals in crop production is needed. In addition, organic farming is widely accepted, and hence alternatives to the use of chemicals are most acceptable and welcome at present. One of the promising alternatives is the use of biological antagonism, which is a safe and sustainable way to manage plant diseases [6,7].

The biological method of protecting plants from pathogens is based on the use of antagonistic microorganisms [8,9]. While biological microbicides were mainly developed from *Bacillus*, *Lactobacillus*, *Pseudomonas*, *Streptomyces, Candida*, *Trichoderma,* and other antagonists previously [10], some underexplored pharmaceutical microorganisms have begun to receive more attention. Usually, these underexplored microbes can produce antibiotics with different structures and diverse pharmacological effects compared with traditional resources [11]. On the other hand, due to the changing environments, biological control has not always produced encouraging results [12]. Thus, strains with better adaptability and field survival should be prospected.

Myxobacteria are ubiquitous Gram-negative bacteria with complicated multicellular morphogenesis and complex social behaviors [13]. The following features make myxobacteria potentially excellent biocontrol agents: (1) myxobacteria are micropredators that actively kill prey cells of both bacteria and fungi to consume their biomass; (2) myxobacteria can produce many different classes of antibiotics, including some of that are rarely found; (3) myxobacteria are capable of forming fruiting bodies filled with myxospores that are responsible for the survival of myxobacteria under unfavorable environmental conditions such as desiccation, high temperature, and UV irradiation; (4) myxobacteria are indigenous soil bacteria and are widely distributed in different types of soil. However, myxobacteria have received less attention than commonly reported antagonistic bacteria described above in agriculture. Few studies have evaluated interactions between myxobacteria and plant pathogens [14,15,16], and the role of myxobacteria in plant health remains largely unknown [14,17]. In isolation and screening of candidate biocontrol myxobacteria, *Myxococcus* sp. strain BS with potential biocontrol activity against bacterial soft rot of calla lily [18] and *Corallococcus* sp. EGB with antifungal activity against cucumber *Fusarium* wilt [19,20] were reported. Studies on myxobacteria functional exploration are necessary for their potential application as biocontrol agents.

The present study reported that a predatory myxobacterium strain M34 isolated from forest soil showed efficient antifungal activities against filamentous pathogenic fungi and lytic activities against Gram-positive and Gram-negative pathogenic bacteria. This promising biocontrol agent was identified as a potential new genus and new species proposed as *Citreicoccus inhibens* gen. nov. sp. nov. The antifungal and lytic activity against phytopathogens revealed the broad-spectrum antimicrobial potential with different strategies of the novel myxobacterium.

## 2. Materials and Methods

### 2.1. Isolation and Purification Process of Myxobacterium Strain M34

Strain M34 was isolated from forest subsoil collected from Nanling National Nature Reserve (N 24°50′22″, E 112°47′23″), Guangdong Province, China. The strain was isolated by standard baiting method using *Escherichia coli* as prey on water agar (WAT; 0.1% CaCl_2_·2H_2_O, 20 mM HEPES, 1.5% agar) supplemented with 25 μg mL^−1^ cycloheximide [21]. In brief, small portions of soil samples were placed adjacent to the *E. coli* spot, and swarming predatory colonies or fruiting bodies were observed after incubation at 30 °C. Then, by cutting the furthest swarm colony edge or fruiting body from soil samples and repeatedly transferring onto fresh VY/2 agar (0.5% dried baker’s yeast, 0.1% CaCl_2_·2H_2_O, 1.5% agar), the strain was finally purified and deposited at Guangdong Microbial Culture Collection Center GDMCC) and Korean Collection for Type Cultures (KCTC).

### 2.2. Morphological Characterization and Identification of Strain M34

Growth properties were assessed at various temperatures (at pH 7.6) and pH values (at 30 °C) on VY/2 agar. Morphogenesis including vegetative cell, fruiting body, spores, and swarm were observed with stereomicroscope, optical microscope, scanning electron microscopy (SEM), and transmission electron microscopy (TEM). The isolate was inoculated on VY/2 agar plates incubated at 30 °C for 5 days. Swarms were carefully scraped and resuspended for observation of morphology, and fruiting bodies were crushed to release myxospores. For SEM, strain M34 were grown on VY/2 agar for 5 days, and 5 mm × 5 mm × 1 mm agar with bacteria cells were carefully cut with sterilized scalpel. The sample was fixed with 3% glutaraldehyde in 0.1 M sodium cacodylate (pH 7.2) for 5 h. After washing 6 times with PBS buffer, the sample was dehydrated in an aqueous alcohol series (30%, 50%, 70%, 90%, and 100%), replaced with tertiary butanol, and lyophilized. Then, the sample was coated using a sputter coater and imaged using a S-4700 SEM microscope (Hitachi, Tokyo, Japan). For TEM, strain M34 were grown in MD1 broth (0.3% Casitone, 0.07% CaCl_2_·2H_2_O, 0.2% MgSO_4_·7H_2_O, trace elements) for 5 days, centrifuged, and washed twice with TPM buffer (10 mM Tris-HCl (pH 7.6), 1 mM KH_2_PO_4_ (pH 7.6), 8 mM MgSO_4_). The pellet was fixed with 3% glutaraldehyde, followed by a secondary fixative of 1% osmium tetroxide. Samples were resuspended in 100 μL 2% agar solution and left at 4 °C overnight before sectioning. Sections were observed using a H7650 TEM microscope (Hitachi, Tokyo, Japan).

Physiology and biochemical characterization of M34 were conducted. Lytic actions toward casein, gelatin, starch, chitin, xylan, lipids (4-nitrophenyl palmitate and 4-nitrophenyl octanoate), cellulose, and skim milk were determined as described [21]. The base agar WAT was overlayed by a thin layer of the described substrates agar (0.5% substrate, 0.1% MgSO_4_·2H_2_O, 0.02% K_2_HPO_4_, 1.5% agar, pH 7.6). The strain M34 suspension was inoculated on the top agar, and the lytic actions were recorded. The predation of M34 to *E. coli* and yeast were conducted as previous report [22]. The fatty acid profile was also included to identify the isolated strain according to Livingstone et al. [22]. Briefly, strain grew on VY/2 agar at 30 °C and pH 7.6. Then, the cellular fatty acids were saponified by incubating in NaOH at 100 °C for 30 min and methylated with HCl in methanol. Fatty acid methyl esters (FEME) were extracted in 1:1 hexane-methyl tert-butyl ether and washed with 0.3 M NaOH. The prepared FEMEs were analyzed using an Agilent 6890N gas chromatograph with a 5973 mass spectrometer (GC–MSD, Palo Alto, CA, USA). The GC was equipped with an Agilent HP-5 capillary column (30 m × 0.32 mm × 0.25 μm). Helium was used as carrier gas with a flow rate of 1.0 mL min^−1^. A split injection (1:10) of 1 μL of sample was used. The column temperature was held at 150 °C for 5 min, ramping at 5 °C min^−1^ to 260 °C and then at 30 °C min^−1^ up to 300 °C for 5 min. The mass operating parameters were as follows: the ion source temperature was 230 °C, and the interface temperature was 150 °C. The total ion current (TIC) spectra were recorded in the mass range of 40–700 *m*/*z* in scanning mode. Data were analyzed using the AMDIS version 2.64. The identification of mass spectra was conducted according to the NIST 14 database. The data presented are mean values of three cultures. The fatty acid profiles of other related *Corallococcus* species for comparison with strain M34 were derived from Livingstone et al. [22].

Isolate M34 was further identified by 16S rRNA gene sequencing with the primers F27 and 1492R [23]. Amplification, sequencing, and phylogenetic analysis of 16S rRNA gene were carried out as described previously [24]. The 16S rRNA gene sequence was compared with the EzTaxon database [25] to identity the most similar species. Phylogenetic tree was constructed using MEGA X [26].

### 2.3. Genomic Sequencing and Bioinformatic Analysis

A swarm of strain M34 was inoculated in MD1 liquid medium, which was then incubated in a shake flask for 5 days at 30 °C. Genomic DNA was extracted from spin-down biomass and sequenced by Majorbio (Shanghai, China) on the Illumina Hiseq 4000 platform using 2 × 250 bp paired-end strategy. The adapter and low-quality reads were filtered by quality control, and the high-quality reads were assembled to contigs using SPAdes 3.13.1 [27]. Contigs underwent CheckM analysis to estimate the genome completeness and degree of contamination [28]. The contigs were then annotated using PROKKA version 1.14.6 [29] and RAST version 2.0 [30]. The whole-genome phylogeny was generated using the TYGS server (https://tygs.dsmz.de (accessed on 9 January 2021)) [31]. In addition, the digital DNA–DNA hybridization (dDDH) values were calculated using the Genome-to-Genome Distance Calculator GGDC 2.1 (http://ggdc.dsmz.de (accessed on 9 January 2021)) [32]. The genomic average nucleotide identity (ANI) was calculated by using the EzBioCloud (www.ezbiocloud.net/tools/ani (accessed on 16 August 2021)) [33]. The genomic taxonomy based on overall genome-relatedness indices ANI and dDDH was also used to identify the isolate, which has been proposed as the new standard for sequence-based taxonomic assignment [34,35]. To further assess whether strain M34 should be assigned to a new species within the *Corallococcus* genus or to a new species of a new genus, average amino acid identity (AAI) values were also used for amino acid-level comparisons based on genomic datasets of proteins using the AAI calculator (http://enve-omics.ce.gatech.edu/aai/ (accessed on 2 October 2021)) [36], which is a sensitive and reliable parameter over greater evolutionary distance to distinguish distantly related populations (such as genera, in this case) [37].

Previous study demonstrated that antibiotic production plays a role in myxobacterial predation [38]. Therefore, we also analyzed the biosynthetic gene clusters (BGCs) of strain M34 using antiSMASH server with version 5.2.0 (https://antismash.secondarymetabolites.org (accessed on 13 January 2021)) [39]. *Myxococcus xanthus* out-membrane vesicles with hydrolase cargo packed have been demonstrated to be directly involved in the lysis of prey cells [40]. Hence, the genes coding carbohydrate active enzymes of strain M34 were determined by using dbCAN2 server (http://bcb.unl.edu/dbCAN2/blast.php (accessed on 19 May 2021)) [41] with e value < 10^−23^ and >80% query coverage, which automatically mines the CAZyme database [42].

### 2.4. Evaluation of the Antagonistic Effect of Strain M34 against Plant Pathogenic Fungi, Gram-Positive and Gram-Negative Bacteria Strains

The preliminary confrontation dual culture (for fungi) and lawn dual culture (for bacteria) were carried out to test the antagonistic and predatory activities of strain M34 against fungi (*Rhizoctonia solani* Kühn, *Fusarium oxysporum* f. sp. cubense race 4, and *Gloeosporium musarum* Cke. and Massee) and Gram-positive (*Clavibacter michiganensis* JCM 1370, *Curtobacterium flaccumfaciens* GIM 1.343, and *Rhodococcus fascians* NBRC 12155) and Gram-negative (*Pseudomonas syringae* GIM 1.330, *Ralstonia solanacearum* GIM 1.70, and *Xanthomonas campestris badrii* JCM 20466) bacteria. For the confrontation dual culture, a 5 mm diameter plug of M34 was placed at 3 cm from the plate (9 cm) edge of freshly prepared potato dextrose agar (PDA) incubated at 30 °C for 3 days; then, a 5 mm diameter plug from an actively growing mycelial culture of fungi was placed at 3 cm from M34. For the lawn dual culture, a spot of 200 μL bacterial suspension (OD_600_ = 5) was inoculated on the center of TPM agar; then, a 5 mm diameter plug strain of M34 was inoculated on the center of the pathogen colony; the plates were incubated at 30 °C. The zones of inhibition or predation were measured to evaluate the antagonistic effect. The growth of fungal hyphae was observed with a stereomicroscope (Olympus SZX10, Olympus Corporation, Tokyo, Japan).

### 2.5. Chemical Analysis of Crude Extract from Strain M34

Strain M34 was inoculated in VY/4 liquid medium (0.25% dried baker’s yeast, 0.1% CaCl_2_·2H_2_O, 1.5% agar) and incubated for 7 days at 30 °C and 180 rpm. At the end of fermentation, 2% XAD-16 adsorber resin was added and incubated in a shaken incubator overnight. The resin was separated by sieving with eight layers of gauze. The separated resin was extracted with methanol for 4 h and sieved with gauze into bottom flasks. At approximately 40 °C, the methanol was evaporated in a rotary evaporator. Finally, the raw extracts were dissolved with H_2_O, and extracted with ethyl acetate and n-butyl alcohol, respectively. The extracted partitions were evaporated, and the three crude extracts were dissolved in 40% dimethyl sulfoxide (DMSO) water solution, respectively. The crude extracts solution was then sterilized with 0.22 μm filters.

The ethyl acetate-soluble partition was first passed through the reversed-phase C18 column via methanol and ultrapure water elution with the ratios of 15%/85%, 35%/65%, 55%/45%, 75%/25%, 90%/10%, and 100%/0. Then, thin-layer chromatography (TLC) was performed to separate the active fraction. A spot of each obtained fraction was dissolved in methanol, loaded on TLC plate, and developed using dichloromethane–methanol (30:1 *v*/*v*) under the solvent vapor saturation condition. Bands were visualized using UV irradiation at 254 nm and stained with the aldehyde reagent. The UV active fractions were purified using preparative high-performance liquid chromatography (HPLC, 40%/60% to 100%/0 acetonitrile–H_2_O over 46 min, 3 mL min^−1^ for compound 1, and 40%/60% to 100%/0 acetonitrile–water over 51 min, 3 mL min^−1^ for compound 2) using a Kromasil 100-5-C18 column (10.0 × 250 mm, 5 μm). The yield and retention time for compounds 1 and 2 were 5.8 mg, 24.5 min and 4.7 mg, 30.8 min, respectively.

The two isolated compounds were subjected to LC/MS analysis using the maXis mass spectrometer (Bruker, Bremen, Germany) interfaced with a LC-20 AT system (Shimadzu, Tokyo, Japan) for recording high-resolution electron spray ionization mass spectrometry data, respectively. Samples were analyzed using the Agilent ZORBAX SB-C18 column (3.0 × 250 mm, 5 μm). Chromatographic conditions for LC/MS analysis were set as follows: flow rate 0.3 mL min^−1^, solvent A (H_2_O with 5 mM acetic acid), and solvent B (CH_3_CN with 5 mM acetic acid). The 1D and 2D NMR spectra were measured on a 600 MHz Avance-III HD spectrometer (Bruker, Bremen, Germany). Chemical shifts were shown in δ values (ppm) relative to CD_3_OD at 3.31 ppm for ^1^H NMR and at 49.0 ppm for ^13^C NMR. For unambiguous assignment of ^1^H and ^13^C signals, 2D NMR experiments (COSY, HSQC, HMBC) were conducted using standard parameter settings and standard pulse programs.

### 2.6. Assessment of the Antagonistic Activities of M34 Crude Extracts, Different Soluble Partitions, and the Purified Compounds against Plant Pathogens

The raw extracts, the ethyl acetate-soluble partition, and the n-butyl alcohol-soluble partition were used to test the antagonistic effect against plant pathogens by using the agar well diffusion method. Briefly, a 5 mm agar plug of fungi pathogen was inoculated to the center of plate, and the 5 mm-diameter well at 2 cm from the pathogen inoculant made by puncher was added with 20 μL of each extract. Geneticin (100 μg well^−1^) and 40% DMSO were used as positive control and negative control, respectively. The plates consisting of three replicates were incubated overnight at 30 °C, and antimicrobial activity was determined by the inhibition of microbial growth around the well. The filter paper disc diffusion method was used to test the antibacterial activities of the crude extracts. Because the lytic activity, rather than antibacterial activity, was observed in the dual culture, we only chose a medium concentration (50 mg mL^−1^) to test the bioactivity. Briefly, 200 μL phytopathogenic bacteria suspension (OD_600_ = 0.1) was spread on nutrient broth plate, and four filter paper discs (5 mm diameter) were placed on the plate. Then, 20 μL crude extract was dropped on each disc. The gentamicin (100 μg mL^−1^) and 40% DMSO were used as positive control and negative control, respectively. Three replicates were set for each treatment. All the plates were incubated at 30 °C overnight, and the bacterial growth was observed to evaluate the antibacterial activity.

As mentioned above, the three fungal species were chosen as targets to test the antagonistic effect of strain M34 because the hyphae grow differently on PDA. *G. musarum* have the most aerial mycelia, followed by *F. oxysporum*, and *R. solani* have the most substrate mycelia. Considering the yields of the isolated compounds described above (NMR and mass spectrometry need much more compounds), we only chose *F. oxysporum* to test the bioactivity of the isolated compounds. The inhibitory activity of purified compounds against *F. oxysporum* was performed with 96-well plates. Spore suspensions were obtained by flooding culture on PDA with 10 mL sterile distilled water, gently swirling the surface with a wire loop to favor detachment of conidia and passing the suspension through four layers of sterile gauze. Then, the centrifuged spore pellets were rinsed twice in sterile distilled water, and the spore concentrations were measured using repeated hemocytometer counts to a final concentration of 1 × 10^4^ spores mL^−1^. The compounds were diluted by the double dilution method, with final concentrations ranging from 0.125 to 256 μg mL^−1^. A mixture of treatment groups with different concentrations (100 μL) and a suspension of the spore suspension (100 μL) were added to each well. Then, 100 μL of spore suspension added with 100 μL dilution solution were used as negative control. The positive control used geneticin (150 μg mL^−1^). Each treatment was conducted in triplicate. The 96-well plates were incubated at 30 °C for 36 h. Turbidity measurement is inaccurate due to the clump growth of *F. oxysporum* in liquid medium, and the hyphae growth under different treatments were observed with naked eyes.

### 2.7. Lytic Activity of Strain M34 Crude Enzymes against Plant Phytopathogens

The strain M34 was cultured in MD1 broth for 5 days at 180 rpm and 30 °C. The supernatant was collected after centrifugation at 12,000 rpm at 4 °C. The supernatant was concentrated by ultrafiltration with the molecular cut-off of 3 kDa. The lytic activity of crude extracellular proteins (1.84 mg mL^−1^ indicated by Braford method) was tested by *Cl. michiganensis* and *G. musarum*. The bacterial pathogen *Cl. michiganensis* was grown in nutrient broth overnight, and then the resuspended cells were adjusted to OD_600nm_ = 0.1 with PBS buffer. Then, 10 μL of cell suspension was spotted to nutrient broth agar. Dried spots were overlayed with 2 μL of crude extracellular protein solution, and the plates were incubated overnight at 30 °C and observed using a stereomicroscope. Lysozyme and Tris-HCl buffer were set as positive and negative control, respectively. Both mycelium and hyphae can act as substrates for the lytic proteins, and the conidia harvest of *G. musarum* on PDA is the most prolific and least time-consuming among the three fungal species. On the other hand, the predatory lysis of strain M34 against the three species was not observed. From a practical purpose, the lytic mechanism of strain M34 as potential biocontrol agent against the three fungal species was unlikely. Therefore, *G. musarum* was used to confirm the lysis of crude proteins. The fungal pathogen *G. musarum* was grown on PDA agar at 28 °C for fresh hyphae collection. A 5 mm-diameter plug from actively growing mycelial culture was placed on a PDA plate, and the hyphae were scraped when the colony diameter reached 2 cm and the collected hyphae were thoroughly resuspended in 10 mL Tris-HCl buffer to avoid hyphae clumps. The fungus was incubated on PDA plate at 28 °C for conidiation collection, as described above, which is a very simple method for the conidia collection of *G. musarum*. The conidia concentration of 1 × 10^4^ in the reaction solution was used. The prepared hyphae and conidia were treated with crude extracellular protein solution for 12 h, and observed by SEM.

## 3. Results

### 3.1. Strain M34 Isolated from Subtropical Forest Soil Exhibited Defining Features of Members from the Myxococcaceae Family

**Figure 1:**
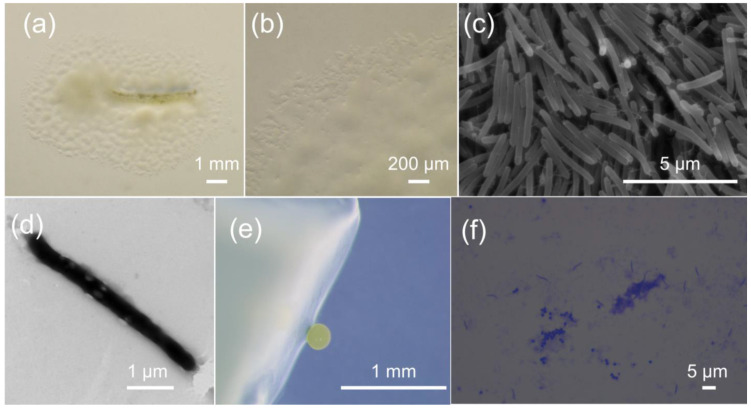
Growth and morphology of strain M34. (**a**) The spreading swarm colony of M34 on VY/2 agar after 1 week of growth. (**b**) Swarm colony edge on VY/2 agar with defined veins and flared edges. The scanning electron micrograph (**c**) and transmission electron micrograph (**d**) of M34 vegetative cells showing slender and flexuous-shaped rods. (**e**) The pale lemon-yellow and roundish fruiting bodies formed on VY/2 agar. (**f**) The crystal violet stained peripheral rods and roundish myxospores from cracked fruiting bodies.

Strain M34 was isolated from the subsoil of a subtropical forest in South China, using the standard *E. coli* baiting method for myxobacteria isolation. The pale lemon-yellow and roundish fruiting bodies appeared at the edge of the soil sample after 7 days of inoculation. Colonies of strain M34 had a transparent to pale lemon-yellow color on VY/2 agar with a thin, film-like appearance (Figure 1a). Distinct veins and flares were observed at the colony border (Figure 1b). At the edge of the swarm border, the cells were composed of slender rods measuring 0.3~0.5 × 2.0~6.0 μm with a mean length of 4~6 μm. The vegetative cells of M34 were interconnected with extracellular fibrils indicated by SEM (Figure 1c). Poles of the cells were rounded, showing similarity to members of Myxococcaceae (Figure 1c,d). The pale lemon-yellow and roundish fruiting bodies (diameter of 200~300 μm) were observed on the VY/2 plate (Figure 1e). The peripheral rods and roundish myxospores from cracked fruiting bodies could be stained by crystal violet (Figure 1f). When grown in liquid culture in VY/2 or MD1, strain M34 grew as spherical clumps. Testing for growth at different temperatures (buffered VY/2 with pH 7.6) demonstrated that M34 grew at a temperature of 25~40 °C, which showed a relatively higher high-temperature tolerance than the closely related species from *Corallococcus* according to Livingstone et al. [22]. The optimum pH of M34 was 7.0, which was similar to other species of *Corallococcus*. Substrate lytic action tests indicated that *E. coli*, skim milk, and starch were efficiently lysed and cleared by M34. The predation to *E coli* and yeast are a common feature of *Corallococcus* spp., *Myxococcus* spp., and other myxobacteria. While 13-methyl-tetradecanoic acid was the major fatty acid of *Corallococcus* spp., the fatty acid profile of M34 was different from that of other *Corallococcus* strains.

**Figure 2:**
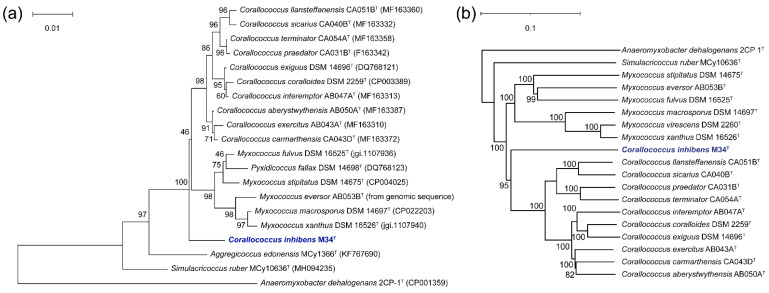
Phylogenetic trees based on 16S rRNA gene sequences (**a**) and genome sequence (**b**) of strain M34 and the closely related type strains. The numbers above branches are pseudo-bootstrap support values from 1000 replicates. The tree was rooted with *Anearomyxobacter dehalogenans* 2CP 1^T^. The strain M34 was noted in blue font.

The complete 16S rRNA gene was retrieved from draft genome sequence with a length of 1536 bp. A BLASTN analysis of the sequence against EzBioCloud 16S rRNA gene database showed the closest similarity to *C. exercitus* AB043A (98.18%), *C. aberystwythensis* AB050A (98.18%), *C. llansteffanensis* CA051B (98.02%), *C. coralloides* DSM 2259 (98.01%), *C. carmarthensis* CA043D (97.94%), *C. exiguus* DSM 14,696 (97.94%), *M. fulvus* DSM 16,525 (97.94%), *C. sicarius* CA040B (97.87%), *M. macrosporus* DSM 14,697 (97.87%), *M. stipitatus* DSM 14,675 (97.87%), and *C. interemptor* CA040B (97.85%), which suggests relatedness to members of *Corallococcus* and *Myxococcus*. The phylogenetic tree based on 16S rRNA gene sequences of M34 and the related species from Myxococcaceae indicated that the isolated strain M34 probably represents a novel genus and novel species of Myxococcaceae (Figure 2a).

The draft genome of strain M34 comprised 9,046,820 bp of sequence with a 69.5% GC content spread over 123 contigs, with an N50 of 208,455 bp and an L50 of 13. The RAST-based annotation of the M34 draft genome identified 7615 protein-coding sequences and 53 RNA genes. The 47.7% predicted proteins were assigned to putative functions by RAST, while 52.3% were hypothetical. Overall, the majority of protein-coding sequences were classified into subsystems of amino acids and derivatives (306), protein metabolism (225), carbohydrates (210), cofactors, vitamins, prosthetic groups, pigments (182), fatty acids, lipids and isoprenoids (151), and membrane transport (107).

The phylogenomic tree, based on TYGS, showing the relationship between strain M34 and the related type strains indicated that the strain M34 shows the highest similarity to the *Corallococcus* clade with high pseudo-bootstrap support values, which is a new clade located in Myxococcaceae (Figure 2b). In silico genome-to-genome comparisons unambiguously showed that strain M34 possessed 19.3~22.4% dDDH and 75.24~79.75% ANI values with other species of Myxococcaceae. The AAI values between M34 and the members of genera *Corallococcus*, *Myxococcus,* and *Simulacricoccus* were 72.57~71.73%, 70.23–70.85%, and 63.31%, respectively. While these AAI values seem among the threshold range of 60~80% for separating different genera [37], the distinct phylogenetic lineage of strain M34 was also supported by lower values than those obtained between the genomes of *Corallococcus* spp. (82.88~93.28%). On the basis of genomic and phylogenetic differences, different growth characteristics, and fatty acid profiles, we propose that the candidate strain described here represents a novel species of a novel genus.

### 3.2. Genome-Based Functional Annotation of Strain M34 Revealed Substantial Antimicrobial and Bacteriolytic Potential

The antiSMASH tool identified 18 complete BGCs and some BGCs on contig edge that might be fragments of BGCs. The complete BGCs include nine NRPS, PKS, and their hybrid gene clusters, seven RRE-containing RiPP clusters (ribosomally synthesized and post-translationally modified peptide; RRE, RiPP recognition element), one thioamitides cluster, and one terpene cluster (Table 1). Many predicted BGCs showing low similarities to known clusters indicated that strain M34 shows substantial biosynthetic potential with potentially novel compounds. For example, Region 1.1 showed 20% similarity to the known leupyrrin clusters (BGC0000380) and was predicted to code the hybrid of NRPS and T1PKS. While Region 1.1 shares three PKS modules with the known leupyrrin clusters, their module numbers of NRPS and modified domains are greatly different, which can be expected in coding novel macrolide compounds.

**Figure 3:**
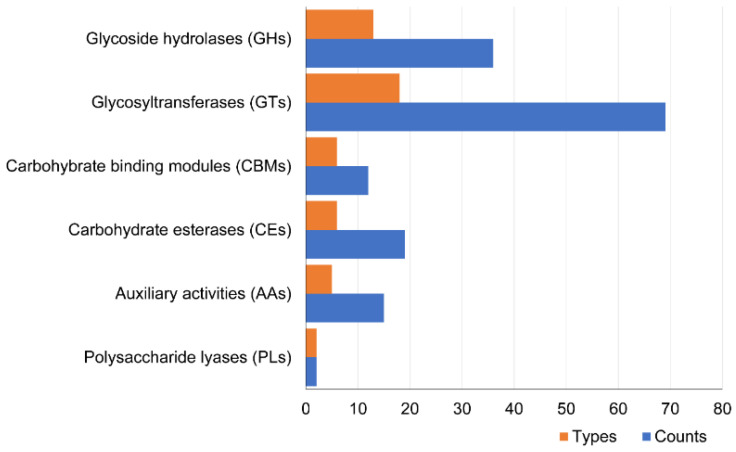
Total CAZymes in the various families, predicted in M34 genome.

The dbCAN analysis of the M34 genome predicted 153 CAZymes responsible for metabolism of various types of carbohydrates and related compounds. The predicted CAZymes were distributed across 50 different CAZymes families, with GTs and GHs constituting the most abundant families in the M34 genome (Figure 3). Carbohydrate-active enzymes belonging to the GHs and PLs families are responsible for metabolism of different biomacromolecules. All the predicted GHs belong to 13 families, and the GH13 and GH23 families constituted the most abundant GH families in M34 genome. Specifically, 10 of 36 GH proteins belonged to the GH13 family, and 9 of 36 GHs were predicted to be in the GH23 family (Figure 3). The abundant GH23 family with predicted lysozyme activity implied the potential lysis against pathogen bacteria.

### 3.3. The Crude Extracts of M34 Fermentation Broth and Isolated Compounds Showed Antifungal Activity against Filamentous Fungi In Vitro

**Figure 4:**
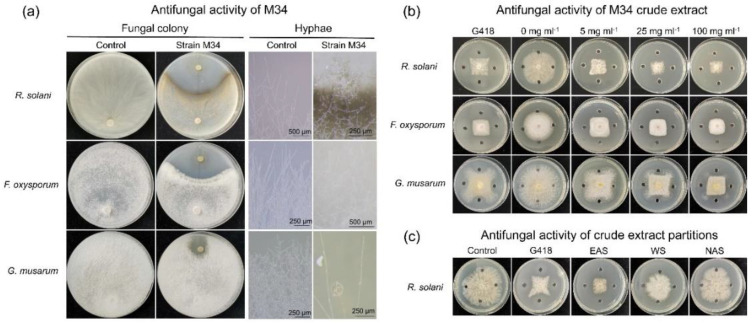
Antifungal activities of strain M34 (**a**), crude extracts (**b**), and partitions (**c**) against plant pathogenic fungi. G418, positive control; EAS, ethyl acetate-soluble partition of M34 extracts; WS, water-soluble partition; NAS, n-butyl alcohol-soluble partition.

The strain M34 was first used to test the antagonistic activities against phytopathogens. The results of a dual-culture assay on PDA demonstrated that *R. solani* and *F. oxysporum* hyphae growth were significantly inhibited by coculture with strain M34 (Figure 4a). However, due to mass of aerial hyphae, M34 showed weak antifungal activity against *G. musarum*. Microscopic observation of pathogenic fungi close to M34 colonies revealed alterations of the hyphae, including excessive (*R. solani*) or less branching (*G. musarum*), disorganized, and disrupted hyphae (Figure 4a). Then, the crude extracts and different soluble partitions were used to test the antifungal activities. Specifically, the raw extracts showed higher activity against *R. solani* than that of *F. oxysporum* and *G. musarum,* and all the tests displayed dosage effect (Figure 4b), which confirmed the antifungal effect of M34. Considering the general antagonism against different filamentous pathogenic fungi, we chose *R. solani* as the representative indicator for the subsequent tests. The ethyl acetate-soluble partition exhibited high antagonistic activity against *R. solani*, but the n-butyl alcohol-soluble and water-soluble partition did not inhibit the hyphae growth (Figure 4c). Therefore, the ethyl acetate extracts were used for the compound isolation; however, the extracts did not show antibacterial activity against the six tested bacteria (Figure S1).

**Figure 5:**
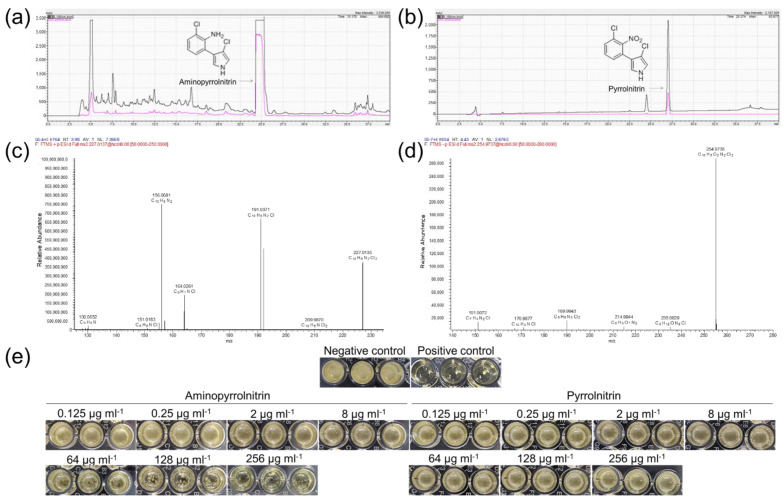
The isolated compounds of strain M34 fermentation broth extracts and their antifungal activity. Fractionation RP-HPLC chromatogram of aminopyrrolnitrin (**a**) and pyrrolnitrin (**c**); HR-ESI-MS spectrum, showing the prominent ion clusters of aminopyrrolnitrin (**b**) and pyrrolnitrin (**d**); the inhibitory activities of compounds against *F. oxysporum* (**e**).

Two fractions obtained from gradient elution (55% methanol/45% H_2_O) of the column chromatography were characterized by TLC and further fractioned and purified by HPLC. Compounds 1 and 2 appeared at retention time 24.5 min and 30.8 min at 190 nm and 245 nm (Figure 5a,b), respectively. Compound 1 was obtained as a pale yellow powder. Its molecular formula was identified as C_10_H_8_N_2_Cl_2_ (*m*/*z* 227.0135 (M+H)^+^) by the HR-ESI-MS ion signal (Figure 5c). The NMR spectral data were as follows: ^1^H-NMR (600 MHz, CD_3_OD) δ: 6.66 (t, *J* = 7.8 Hz, H-5′), 6.80 (d, *J* = 2.3 Hz, H-5), 6.85 (d, *J* = 2.3 Hz, H-2), 7.02 (dd, *J* = 1.5, 7.8 Hz, H-6′), 7.16 (dd, *J* = 1.5, 7.8 Hz, H-4′); ^13^C-NMR (150 MHz, CD3OD) δ: 112.0 (C-3), 117.3 (C-2), 118.3 (C-5), 118.7 (C-5′), 119.5 (C-4), 120.2 (C-3′), 122.5 (C-1′), 129.0 (C-6′), 131.2 (C-4′), and 143.3 (C-2′). Compound 1 was identified as aminopyrrolnitrin by NMR spectroscopy and mass spectroscopy data analysis. Compound 2 was a pale yellow powder with the molecular formula of C_10_H_6_N_2_O_2_Cl_2_ (m/z 254.9733 (M-H)^−^) (Figure 5d). The NMR spectral data were as follows: ^1^H-NMR (600 MHz, CD3OD) δ: 6.75 (d, *J* = 2.3 Hz, H-5), 6.84 (d, *J* = 2.3 Hz, H-5), 7.53 (m, 3H, H-4′, H-5′, H-6′); ^13^C-NMR (150 MHz, CD_3_OD) δ: 111.8 (C-3), 115.7 (C-4), 117.9 (C-2), 118.6 (C-5), 125.3 (C-3′), 129.4 (C-1′), 129.6 (C-6′), 131.4 (C-5′), 131.9 (C-4′), and 150.6 (C-2′). Compound 2 was identified as pyrrolnitrin based on the NMR spectroscopy and mass spectroscopy data analysis. The two compounds showed antifungal activity against *F. oxysporum*. Both Compound 1 and Compound 2 inhibited fungal growth with all the tested concentrations (0.125~256 μg mL^−1^), with the latter showing a stronger effect than that of the former (Figure 5e).

### 3.4. Strain M34 and the Extracellular Crude Proteins Showed Lytic Activity against Phytopathogens In Vitro

**Figure 6:**
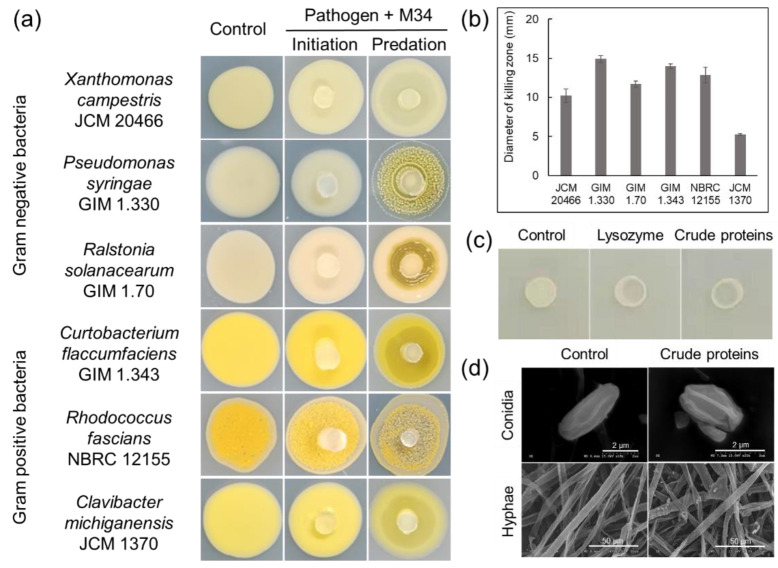
The lytic activity of strain M34 and the extracellular crude proteins against plant pathogens. (**a**) The predation of M34 against plant pathogenic bacteria; (**b**) predatory activity (diameter of killing zone) of strain M34 against Gram-negative and Gram-positive plant pathogenic bacteria; (**c**) lysozyme as positive control; (**d**) SEM showing no obvious lytic effect of extracellular crude proteins on spores and hyphae of *G. musarum*.

Strain M34 was able to feed on all the tested plant pathogenic bacteria in the plate assay, resulting in lytic zone of bacterial colonies overlaid by the growing cells of M34. However, pathogenic bacteria exhibited different susceptibility to M34 with highest predatory activity against *P. syringae* and weakest predatory activity against *Cl. michiganensis* (Figure 6a,b). Intriguingly, predation of two Gram-negative bacteria (*P. syringae* and *Ra. solanacearum*) induced aggregation and rippling behavior of M34 group cells (Figure 6a). Furthermore, our results indicated that the concentrated extracellular protein showed considerable lytic activity against *Cl. michiganensis* JCM 1370 (Figure 6c). However, SEM analysis showed that the treated conidia and hyphae of *G. musarum* stayed intact, which was the same as the buffer-treated control (Figure 6d).

## 4. Discussion

Myxobacteria are soil indigenous bacteria, and most of them can prey on both fungi and bacteria [43]. Predatory bacteria have been proposed as potential biological agents and have shown effective biocontrol activity against soilborne pathogens [44,45,46]. Mining of new microbial resources adapted to complicated soil environments and equipped with different biocontrol mechanisms is necessary for successful biocontrol of phytopathogens. *Myxococcus* and *Corallococcus* are among the most frequently isolated myxobacteria genera from soil [47]. Some *Myxococcus* strains have been evaluated as antimicrobial potentials, especially *M. xanthus* as model species of myxobacteria [48]. Compared to *Myxococcus*, other genera of Myxococcaceae are poorly understood. The genus *Corallococcus* includes 10 species, eight of which were described recently [49]. Here, we provided a new strain, closely related to *Corallococcus,* with potential biocontrol activity against many phytopathogens. Genome sequence-based phylogenetic analysis and the low values of genome-based indices (dDDH, ANI, and AAI) indicated that M34 is highly divergent from the described *Corallococcus* genus, justifying the assignment of M34 to a novel species of a novel genus. Although the myxobacterial colony and fruiting body morphology are important indicators for myxobacteria taxonomic classification, gene- and genome-based approaches are more repeatable and independent of laboratory conditions such as batch variations in media and adaption of lineages to repeated subculturing, which can differ the myxobacteria morphological characteristics [50]. The mechanisms involved in the biocontrol of plant diseases by myxobacteria have not been fully elucidated [14,17,44]. However, several lines of evidence suggest that they exert antagonistic effects toward phytopathogens by predation and antibiosis. For example, *Corallococcus* sp. EGB can prey on pathogenic fungi *Magnaporthe oryzae* and control rice blast disease, with a novel outer membrane beta-1,6-glucanase identified as a promising target to hydrolyze cell walls of pathogenic fungi [51]. The strain M34 was demonstrated to adopt contrasting antagonistic strategies to inhibit plant pathogenic fungi and bacteria. The metabolites in fermentation broth inhibited the growth of filamentous pathogenic fungi, but not plant pathogenic bacteria. The strain M34 and the extracellular crude proteins can lyse cells of plant pathogenic bacteria, but not fungal hyphae and conidia. Our results, combined with previously published reports, revealed that members of Myxococcaceae can be used as potential biocontrol agents to control different kinds of plant diseases with different strategies.

The direct attack of a biocontrol agent against phytopathogens was launched by the antagonistic weapons, including antibiotics and lytic enzymes [8,9]. Investigation of the antagonism biological basis of the strain M34 revealed that the strain can produce pyrrolnitrin, a potent and promising antifungal agent produced by some species of *Pseudomonas*, *Serratia*, *Burkholderia,* and some certain strains of myxobacteria [52,53,54,55]. Previous studies indicated that the antagonistic mechanism of pyrrolnitrin included impeding synthesis of key biomolecules (DNA, RNA, and protein), uncoupling with oxidative phosphorylation, inhibiting mitotic division [55,56]. Pyrrolnitrin has emerged as a lead molecule of agricultural importance, with two synthesized derivatives of pyrrolnitrin registered as agricultural fungicides [55]. Although chemical synthesis of pyrrolnitrin has been obtained using different steps, microbial production is still an easier and more eco-friendly option than chemical synthesis. Therefore, efficient producer strains still need to be explored and characterized. On the other hand, other bioactive secondary metabolites remaining to be detected may also contribute to the antagonism biological basis of the strain M34, which is included in our future work of this strain.

The direct lysis of pathogenic cells by extracellular enzymes is another biocontrol mechanism against phytopathogens [57,58]. Myxobacteria are micropredators feeding on prey cells, which involves the lysis of prey cells with hydrolase cargo loaded by outer-membrane vesicles [17,40]. The lytic enzymes produced by myxobacteria have been shown to promote the control of plant diseases [51]. Strain M34 can grow on the medium supplied with phytopathogenic bacteria as the only carbon and nitrogen source. Furthermore, the lytic activity of crude protein solution evidenced the lytic mechanism of M34 against phytopathogens. This study provides a new microbial source for the identification and exploration of lytic enzymes to control phytopathogens. However, the purification and identification of effective enzymes from M34 have failed due to the low production of proteins under the used fermentation condition. The synergistic effect of several different enzymes to digest pathogen bacteria, rather than only one enzyme, is also an important strategy adopted by strain M34 to effectively lyse bacteria [59].

Furthermore, the most important factors of a successful biocontrol agent also include effective colonization in different environments [60,61]. The effective and long-lasting colonization of biocontrol agents has a direct contribution to successful biological management of soilborne disease. The shared common feature of this native soil myxobacterium to other myxobacteria, such as fruiting body formation under unfavorable conditions, contributes to effective colonization in different changing soil environments, convenient transport, and long-term storage of microbial agents [62,63]. The formation of biofilm structure due to abundant extracellular matrices also facilitates long-lasting myxobacteria colonization in the rhizosphere [20]. In addition, myxobacteria are facultative predators with the ability of assimilating both macromolecules (such as organic matter, dead cells) and live prey cells [17], which shows a broader nutrient source than some other traditional biocontrol agents. These distinct features make M34 and other myxobacteria ideal agents to colonize different soils. However, the next step for the preparation of strain M34 as a biocontrol agent is the application in the field, establishing adequate dosage and evaluating effect of disease suppression.

i. Description of *Citreicoccus* gen. nov.

*Citreicoccus* (Ci.tre.i.coc.cus. L. neut. adj. *citreium*, lemon; N.L. masc. n. *coccus*, coccus; N.L. masc. n. *Citreicoccus*, a lemon-colored coccus).

Vegetative cells are rod-shaped with rounded cell poles. They form a transparent to pale lemon-yellow color on VY/2 yeast agar with a thin, film-like appearance. Distinct veins and flares were observed at the colony border. Fruiting bodies are pale lemon-yellow and roundish, composed of peripheral rods and roundish myxospores. Major cellular fatty acids are the branch-chain type. Yeast, *E. coli*, skim milk, and starch are lysed, but not cellulose, chitin, xylan, or agar. Delineation was determined by 16S rRNA gene sequence phylogeny, genomic phylogeny, and physiochemical and morphological characteristics. The type species is *Citreicoccus inhibens*.

ii. Description of *Citreicoccus inhibens* sp. nov.

*Citreicoccus inhibens* (in.hi’bens. L. part. adj. *inhibens*, inhibiting, referring to the ability of the bacterium to inhibit fungal growth).

In addition to the characteristics of the genus, the vegetative cells are 0.3~0.5 × 2.0~6.0 μm in size. The diameter of pale lemon-yellow and roundish fruiting bodies is 200~300 μm. Vegetative cells are interconnected with extracellular fibrils. The swarm colony varies from transparent to pale lemon-yellow color on VY/2 agar with a thin, film-like appearance and distinct veins and flares at the colony border. Cell aggregates are beige to lemon on VY/2 agar. Optimal growth temperature is 30 °C. Optimal pH is 7.0. The best nutritional sources are medium with peptones or starch. Major fatty acids are iso-C15:0, iso-C16:0, iso-C17:0, and C17:0 cyclo.

The type strain is M34^T^ (=GDMCC 1.2275^T^ = KCTC 82453^T^), isolated from the subsoil of a subtropical forest in South China. The DNA G+C content of the type strain is 69.3%, calculated from its genome sequence. The 16S rRNA gene sequence and genome sequence accession numbers of strain M34^T^ IN GeneBank are MZ407560.2.3 and JAHNZT000000000, respectively.

## Figures and Tables

**Table 1:** Antibiotics and secondary metabolite analysis of complete biosynthetic gene clusters in strain M34.

	Region	Type	Most Similar Gene Cluster	Similarity (%)
Scaffold1	1.1	NRPS ^1^, T1PKS ^2^ hybrid	leupyrrin	20%
Scaffold1	1.2	NRPS	VEPE/AEPE/TG-1	100%
Scaffold2	2.1	NRPS, T1PKS hybrid	myxoprincomide-c506	66%
Scaffold2	2.2	RiPP ^3^ (RRE-containing ^4^)	-	-
Scaffold3	3.1	thioamitides	-	-
Scaffold3	3.2	NRPS, T1PKS hybrid	nostopeptolide A2	50%
Scaffold5	5.1	RiPP (RRE-containing)	-	-
Scaffold6	6.1	RiPP (RRE-containing)	-	-
Scaffold11	11.1	RiPP (RRE-containing)	-	-
Scaffold14	14.1	RiPP (RRE-containing)	-	-
Scaffold16	16.1	T1PKS, NRPS hybrid	DKxanthene	82%
Scaffold17	17.1	T1PKS	-	-
Scaffold18	18.1	NRPS	chloromyxamide	13%
Scaffold23	23.1	RiPP (RRE-containing)	-	-
Scaffold24	24.1	terpene	carotenoid	100%
Scaffold26	26.1	T3PKS ^5^	alkylpyrone-407/alkylpyrone-393	54%
Scaffold42	42.1	T1PKS	sporolide A/sporolide B	10%
Scaffold47	47.1	RiPP (RRE-containing)	-	-

^1^ Non-ribosomal peptide synthetase. ^2^ Type I polyketide synthase. ^3^ Ribosomally synthesized and post-translationally modified peptide. ^4^ RiPP recognition element (domains that can identify and bind the RiPP precursor peptide). ^5^ Type Ⅲ polyketide synthase.

## Data Availability

The 16S rRNA gene sequence of M34 was deposited in NCBI with the accession number of MZ407560. The draft assembly of M34 was publicly available with the accession number of JAHNZT000000000 at NCBI GenBank. Other datasets generated during the current study are available from the corresponding author.

## Supplementary Materials

The following are available online at www.mdpi.com/article/10.3390/microorganisms9102137/s1, Figure S1: The crude extracts of strain M34 did not show antibacterial activity against different phytopathogenic bacteria.
