# Supplementary material for: The Predatory Myxobacterium Citreicoccus inhibens gen. nov. sp. nov. Showed Antifungal Activity and Bacteriolytic Property against Phytopathogens
Source: Microorganisms. 2021 Oct 12;9(10):2137. doi: 10.3390/microorganisms9102137 (PMC8538283; doi:10.3390/microorganisms9102137)
Supplement: Supplementary file 1 [file microorganisms-09-02137-s001.zip › microorganisms-1365972-supplementary.pdf]

microorganisms

MDPI

Supplementary Material

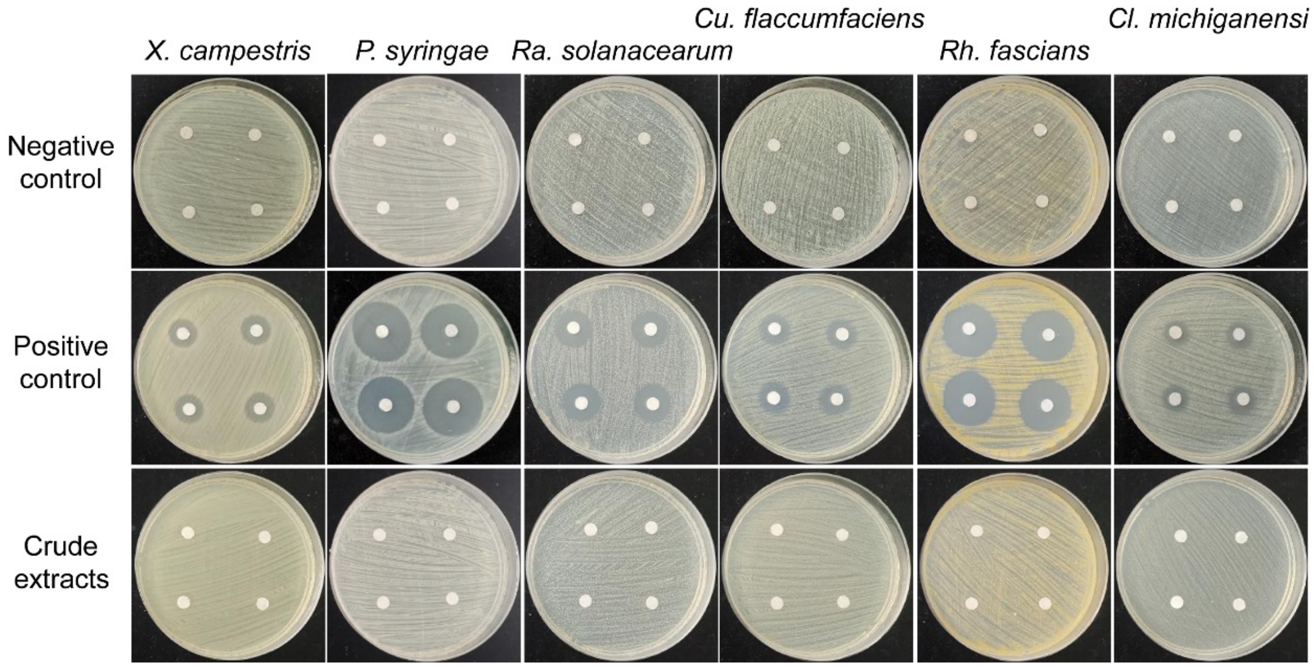

Figure S1. The crude extracts of strain M34 did not show antibacterial activity against different phytopathogenic bacteria.
